# Lenvatinib plus pembrolizumab for systemic therapy-naïve and -experienced unresectable hepatocellular carcinoma

**DOI:** 10.1007/s00262-022-03185-6

**Published:** 2022-03-28

**Authors:** Chi-Jung Wu, Pei-Chang Lee, Ya-Wen Hung, Chieh-Ju Lee, Chen-Ta Chi, I-Cheng Lee, Ming-Chih Hou, Yi-Hsiang Huang

**Affiliations:** 1 Division of Gastroenterology and Hepatology, Department of Medicine, Taipei Veterans General Hospital, 201 Shih-Pai Road, Sec. 2, Taipei, 11217 Taiwan; 2 Institute of Clinical Medicine, National Yang Ming Chiao Tung University School of Medicine, Taipei, Taiwan; 3 Faculty of Medicine, National Yang Ming Chiao Tung University School of Medicine, Taipei, Taiwan

**Keywords:** HCC, Immunotherapy, Target therapy, First-line setting, Systemic therapy

## Abstract

**Background:**

Lenvatinib combined with pembrolizumab showed a promising result in an early phase study for hepatocellular carcinoma (HCC). The efficacy and safety of lenvatinib plus pembrolizumab for patients with unresectable HCC (uHCC) beyond the first-line setting were unclear.

**Methods:**

Seventy-one consecutive patients who received lenvatinib plus pembrolizumab for uHCC were prospectively enrolled. Effect of lenvatinib combinations on Albumin-Bilirubin (ALBI) score and factors associated with progression-free survival (PFS) and overall survival (OS) were analyzed.

**Results:**

Of the 71 cases, 58 (81.7%) were in BCLC C. There were 44 (62%) for the first-line systemic treatment, and 27 (38%) had experienced targeted therapy or nivolumab treatment. The objective response rate and disease control rate (DCR) were 34.1% and 84.1% for the first-line setting, and 18.5% and 70.4% for systemic therapy-experienced cases (Response Evaluation Criteria in Solid Tumors version 1.1, RECIST v1.1), respectively. The mean ALBI score was stable during the treatment course. After a median of 9.3 months of follow-up, the median PFS was 9.3 months versus 4.4 months, and the median OS was not estimable yet versus 12 months for Child–Pugh A versus B patients, respectively. Prior nivolumab failure was the only significant factor associated with poorer PFS (HR = 3.253, *p* = 0.004). Child–Pugh class B (HR = 2.646, *p* = 0.039) and prior nivolumab failure (HR = 3.340, *p* = 0.014) were independent factors for poorer OS in multivariate analysis.

**Conclusions:**

A high DCR was observed by lenvatinib/pembrolizumab combination without adverse effect on ALBI score for systemic therapy-naïve and -experienced uHCC. Suboptimal response to prior nivolumab-failed patients requires further exploration*.*

**Supplementary Information:**

The online version contains supplementary material available at 10.1007/s00262-022-03185-6.

## Introduction

Hepatocellular carcinoma (HCC) is the most common primary malignancy of the liver and ranks as the fourth leading cause of cancer-related mortality in the world [1]. Early stage HCC can be curatively treated by surgery, transplantation, or ablation [2]. Locoregional treatment by transarterial chemoembolization (TACE) is the standard of care for intermediate-stage HCC, but the complete response rate is low [3, 4]. For patients with advanced HCC, systemic treatment by sorafenib can only provide a limited response [5–7].

In recent years, several antiangiogenic multikinase inhibitors (MKI) or antibody and immunotherapy by immune checkpoint inhibitors (ICIs) have become available for advanced HCC [5, 6, 8–13]. Nivolumab and pembrolizumab are both ICIs that block programmed death 1 (PD-1) and have shown response rates of 17–20% in phase I/II studies [12, 13]. The phase III study of pembrolizumab in a second-line setting failed to reach a prespecified p-value, but clinically meaningful outcomes were observed [14].

Vascular endothelial growth factor (VEGF) inhibitor could enhance antitumor immunity restored by PD-1/PD-L1 antibody by antagonizing the VEGF-mediated immunosuppressive effect within the tumor microenvironment [15, 16]. Promising results with survival benefits have been confirmed for atezolizumab plus bevacizumab in a phase III trial for HCC [17]. Currently, there are multiple phase III clinical trials being conducted to examine combination therapies of ICI plus MKI or ICI plus ICI. These combinations include lenvatinib plus pembrolizumab, atezolizumab plus cabozantinib, durvalumab plus tremelimumab, and nivolumab plus ipilimumab in a first-line setting [10, 18–21]. Among this list, lenvatinib plus pembrolizumab has shown promising data for the response rate and survival in the phase 1b study [18].

Previous clinical trials excluded patients with high-risk tumors (tumor volume ≥ 50% of the liver volume, main portal vein invasion, or biliary tract invasion) and decompensated liver function [8, 18]. Thus, it remains unclear whether such patients could respond to lenvatinib plus pembrolizumab, and safety profiles should be further delineated. Therefore, this study reports the treatment responses, risks of treatment-related adverse effects (TRAEs), albumin-bilirubin (ALBI) score changes [22], and survival in response to lenvatinib plus pembrolizumab in the first-line setting or in cases where prior systemic treatment failed in patients with unresectable HCC. Furthermore, we also identify predictors associated with survival.

## Materials and methods

### Patients

This study prospectively enrolled consecutive 71 patients with unresectable HCC who received lenvatinib plus pembrolizumab from July 2019 to February 2021 at Taipei Veterans General Hospital. Both lenvatinib and pembrolizumab had been approved for HCC by the Taiwan Food and Drug Administration (TFDA) before this study began. The key inclusion criteria included age > 20 years; diagnosis of HCC confirmed by pathologic or radiographic features according to the American Association for the Study of Liver Diseases (AASLD) criteria; and HCC status of Barcelona Clinic Liver Cancer (BCLC) stage B or C not amenable or refractory to TACE. High-risk tumors were allowed in this study, including tumors with size ≥ 50% of the liver size, main portal vein invasion (Vp4), or biliary tract invasion.

The exclusion criteria were patients who had concurrent cancer other than HCC, hepatic encephalopathy, severe comorbidities (end-stage renal disease, acute coronary syndrome, cerebrovascular accident, severe heart failure, severe arrhythmia, and severe trauma), Child–Pugh class C, and underlying autoimmune diseases. The dose of lenvatinib complied with the standard body weight (BW)-based recommendation (12 mg for BW ≧ 60 kg, 8 mg for BW < 60 kg, orally once daily). Pembrolizumab was administered intravenously at 200 mg or 2–3 mg/kg every 3 weeks [23–25].

This study was approved by the Institutional Review Board of Taipei Veterans General Hospital (IRB number: 2020–06-033BC, 2021–07-040BC). The study complied with the standards of the Declaration of Helsinki and current ethical guidelines. All patients signed informed consent forms.

### Assessment and follow-up

For this prospective study, all patients had a uniform protocol for monitoring and assessment. Tumor assessment imaging (computed tomography, magnetic resonance imaging, or both) was performed before starting the treatment and repeated every 9 weeks. Serum alpha-fetoprotein (AFP) was measured every 3 weeks until image-confirmed progression occurred. Early AFP response (> 10%) for patients with baseline AFP ≥ 10 ng/mL was defined as a reduction of AFP > 10% within 4 weeks of the treatment [25]. Any AFP response for patients with baseline AFP ≥ 10 ng/mL was defined as any degree of AFP reduction within 4 weeks of the treatment. The response was assessed according to the Response Evaluation Criteria in Solid Tumors version 1.1 (RECIST v1.1) [26] and hepatocellular carcinoma-specific modified RECIST (mRECIST) [27]. TRAEs were graded according to the National Cancer Institute Common Terminology Criteria for Adverse Events (version 5.0).

### End points

The primary end point was the tumor response, including the objective response rate (ORR) and disease control rate (DCR) evaluated by RECIST v1.1 and mRECIST. The secondary end points were progression-free survival (PFS), overall survival (OS), time to response (TTR), time to progression (TTP, defined as time from starting treatment to the first progressive disease (PD)), duration of response (DOR, defined as time from the first partial response (PR) or complete response (CR) to PD or death), and incidence of TRAEs.

### Clinical features, biochemistry tests, serological markers and PD-L1 expression analysis

The following clinical features and biochemical data were collected for analysis: age, sex, BCLC stage, Child–Pugh score, serum AFP, alanine aminotransferase (ALT), aspartate aminotransferase (AST), creatinine, albumin, total bilirubin levels, platelet count, and prothrombin time (international normalized ratio (INR)). Serum biochemistry tests were done using a systemic multi-autoanalyzer (Technicon SMAC, Technicon Instruments Corp., Tarrytown, NY). Serum AFP was measured using radio-immunoassay kits (Abbott Laboratories, North Chicago, IL and Serono Diagnostic SA, Coinsin/VD, Switzerland, respectively).

PD-L1expression was measured by an immunohistochemistry pharmDx assay (Agilent Technologies, Santa Clara, CA, USA) with anti-PD-L1 22C3 antibody on archival or newly obtained biopsy HCC tissues [25]. Expression levels are reported as the tumor proportion score (TPS) and combined positive score (CPS) [28]. TPS or CPS ≧ 1% was defined as a positive result.

### Statistical analysis

Categorical data were compared using Fisher’s exact test or Pearson’s chi-squared analysis. ORRs were calculated with the 95% confidence interval (CI) using the Clopper-Pearson method. For patients with confirmed PR or CR, DOR and TTR were analyzed. The changes in ALBI score were estimated by a linear mixed model, which can be used to analyze an outcome measured repeatedly from the same individuals. The Kaplan–Meier method with the log-rank test was applied for OS, PFS, TTP, and DOR.

Variables that achieved statistical significance (p < 0.05) or were close to significance (p < 0.10) in the univariate analysis were examined in a multivariate analysis. For all analyses, p < 0.05 was considered statistically significant. All statistical analyses were performed using the Statistical Package for Social Sciences (SPSS 28.0 for Windows, SPSS Inc, Chicago, IL) and GraphPad Prism version 9.1 (GraphPad Software, La Jolla, CA, USA).

## Results

### Patient characteristics

The baseline characteristics of the 71 HCC patients are listed in Table 1. The median age was 63 years, and 87.3% of the patients were male. Most patients were within Child–Pugh class A (*n* = 51, 71.8%), ECOG performance status of 0/1 (*n* = 62, 87.3%), and BCLC C (*n* = 58, 81.7%). The etiology of HCC was predominantly hepatitis B virus (HBV) (63.4%).

**Table 1:** Baseline characteristics of the 71 HCC patients

Parameter	Entire cohort	First-line setting	Systemic therapy-experienced	*p* value
	(*n* = 71)	(*n* = 44)	(*n* = 27)	
Median age, years (range)	63 (28–89)	62 (38–85)	69 (28–89)	0.305
Male, *n* (%)	62 (87.3)	36 (81.8)	26 (96.3)	0.139
ECOG (0/ 1/ 2), *n* (%)	43 (60.6)/ 19(26.8)/ 9(12.7)	30 (68.2)/10(22.7)/4(9.1)	13 (48.1)/9(33.3)/5(18.5)	0.225
BMI(kg/m2), median ± SD	23.23 ± 4.23	23.68 ± 4.90	23.05 ± 2.81	0.33
Child–Pugh class (A/B), *n* (%)	51 (71.8)/ 20(28.2)	33 (75)/11(25)	18 (66.7)/9(33.3)	0.588
ALBI grade (1/ m2a/ m2b/ 3), *n* (%)	28 (39.4)/15(21.1)/25(35.2)/3(4.2)	22 (50)/9(20.5)/13(29.5)/0(0)	6 (22.2)/6(22.2)/12(44.4)/3(11.1)	0.026
FIB-4 score, median (range)	4.33 (0.77–31.68)	3.56(0.77–11.94)	4.62(1.61–31.68)	0.181
BCLC stage (B/C), *n* (%)	13 (18.3)/58(81.7)	8 (18.2)/36(81.8)	5 (18.5)/22(81.5)	1
Etiology (HBV/ HCV^a^/ Other), *n* (%)	45 (63.4)/11(15.5)/18(25.4)	28 (63.6)/5(11.4)/12(27.3)	17(63)/6(22.2)/6(22.2)	0.635^d^
Portal vein invasion, *n* (%)	41 (57.7)	27 (61.4)	14 (51.9)	0.431
Main portal vein invasion (Vp4), *n* (%)	15 (21.1)	11 (25)	4 (14.8)	0.307
Biliary tract invasion, *n* (%)	2 (2.8)	1 (2.3)	1 (3.7)	1
Extrahepatic metastasis, *n* (%)	32 (45.1)	17 (38.6)	15 (55.6)	0.164
Multiple tumors, *n* (%)	50 (70.4)	29(65.9)	21(77.8)	0.287
Tumor volume, ≥ 50% liver volume, *n* (%)	17 (23.9)	12 (27.3)	5 (18.5)	0.401
Serum AFP level, > 400 ng/ml, *n* (%)	32 (45.1)	21 (47.7)	11 (40.7)	0.566
*Prior systemic treatment, n (%)*				
Nil	44 (62)	44 (100)	0 (0)	–
Sorafenib	15 (21.1)	0 (0)	15 (55.6)	
Sorafenib followed by regorafenib	8 (11.3)	0 (0)	8 (29.6)	
Lenvatinib	2 (2.8)	0 (0)	2 (7.4)	
Nivolumab ± MKI^b^	9 (12.7)	0 (0)	9 (33.3)	
N/L ratio, median ± SD	3.52 ± 4.79	4.81 ± 6.65	2.96 ± 4.50	0.018
AST (U/L), median ± SD	56 ± 68	56 ± 72	56 ± 63	0.914
ALT (U/L), median ± SD	39 ± 63	38 ± 44	42 ± 85	0.322
PT (INR), median ± SD	1.18 ± 0.14	1.18 ± 0.12	1.18 ± 0.17	0.324
Platelet count (10^9^ /L), median (range)	139 (27–610)	151 (44–610)	119 (27–451)	0.324
Creatinine (mg/dl), median ± SD	0.88 ± 0.76	0.82 ± 0.83	0.91 ± 0.65	0.799
PD-L1 expression, CPS ≥ 1%, *n* (%)^c^	18 (37.5)	13 (41.9)	5 (29.4)	0.391

*AFP* alpha-fetoprotein, *BCLC* barcelona clinic liver cancer, *CPS* combined positive score, *ECOG* eastern cooperative oncology group, *HBV* hepatitis B virus, *HCV* hepatitis C virus, *N/L ratio* neutrophil-to-lymphocyte ratio, *PD-L1* programmed death ligand 1. *SD*, standard deviation

^a^3 patients with HBV-HCV co-infection

^b^7 patients received both nivolumab and MKIs before and 2 patients received nivolumab only

^c^PD-L1 data were available in 48 patients

^d^Compare viral hepatitis and non-viral hepatitis

There were 44 (62%) patients who were treated in the first-line setting, while the other 27 (38%) patients had experienced prior systemic therapy including MKIs or ICI (sorafenib (*n* = 15), sorafenib followed by regorafenib (*n* = 8), lenvatinib (*n* = 2), nivolumab (*n* = 9)). There were 28 patients who had high-risk tumors, including tumor volume ≧ 50% of the liver volume (*n* = 17), main portal vein invasion (*n* = 15), and biliary tract invasion (*n* = 2). Compared with patients who received prior systemic therapy, patients in the first-line setting had a higher proportion with ALBI grade 1 and higher N/L ratios.

### Tumor response

During a median follow-up duration of 9.3 months (range, 0.7 to 17.8 months), CR was achieved in 1 (1.5%) patient according to RECIST v1.1 and 4 (5.6%) patients according to mRECIST. The ORR was 28.2% (95% CI, 17.5% to 38.9%) according to RECIST v1.1 and 57.7% (95% CI, 46% to 69.5%) according to mRECIST. DCR was 78.9% (95% CI, 69.1% to 88.6%) according to RECIST v1.1 and 80.3% (95% CI, 70.8% to 89.8%) according to mRECIST (Table 2).

**Table 2:** Best objective response evaluated by RECIST 1.1 and mRECIST in HCC patients in the first-line setting or with prior systemic therapy failure

Parameter, *n* (%)	Entire cohort (*n* = 71)		RECIST v1.1			mRECIST		
	RECIST v1.1	mRECIST	First-line setting	Systemic therapy-experienced	*p* Value	First-line setting	Systemic therapy-experienced	*p* Value
			(*n* = 44)	(*n* = 27)		(*n* = 44)	(*n* = 27)	
CR	1 (1.5)	4 (5.6)	1 (2.3)	0 (0)	1	3 (6.8)	1 (3.7)	1
PR	19 (28.4)	37 (52.1)	14 (31.8)	5 (18.5)	0.219	26 (59.1)	11 (40.7)	0.133
SD	36 (53.7)	16 (22.5)	22 (50)	14 (51.9)	0.88	9 (20.5)	7 (25.9)	0.592
PD	15 (21.1)	14 (19.7)	7 (15.9)	8 (29.6)	0.737	6 (13.6)	8 (29.6)	0.1
ORR	20 (28.2)	41 (57.7)	15 (34.1)	5 (18.5)	0.157	29 (65.9)	12 (44.4)	0.075
DCR	56 (78.9)	57 (80.3)	37 (84.1)	19 (70.4)	0.169	38 (86.4)	19 (70.4)	0.1
*For responders*								
Median TTR, months (range)	2.3 (1.8–8.6)	2.17 (1–8)	2.4 (1.8–8.6)	2.3 (1.93–4.13)	0.117	2.2 (1–8)	2.03 (1–3)	0.09
Estimate DOR, months (95% CI)	9.3 (NE)	7.0 (3.4–10.6)	7.0 (4.4–9.5)	NE	0.13	9 (3.6–14.4)	6.8 (0–15.4)	0.612

*CR* complete response, *DCR* disease control rate, *DOR* duration of response, mRECIST modified RECIST, *ORR* objective response rate, *PD* progressive disease, *PR* partial response, *SD* stable disease, *TTR* time to response, *NE* not estimable

For the responders, the median TTR was 2.3 months (range, 1.8 to 8.6) according to RECIST v1.1. The median DOR according to RECIST v1.1 was 9.3 months (95% CI, NE) (Table 2). The ORR for the first-line setting was 34.1%, which is numerically higher than the result of 18.5% for cases that had experienced prior systemic therapy (RECIST v1.1). Compared with the first-line setting, numerically lower ORR and DCR were observed in patients with prior systemic therapy according to both RECIST v1.1 and mRECIST (Table 2). For patients with only MKI experience, the ORR and DCR were 27.8% (vs. 34.1%, *p* = 0.629) and 83.3% (vs. 84.1%, *p* = 1.000) compared with those in the first-line setting (RECIST v1.1), respectively.

The ORR for the patients with high-risk tumors was numerically lower than that of patients with low-risk tumors (25% vs. 30.2%, *p* = 0.632). The DCR was similar between the two groups (85.7% vs. 74.4%, *p* = 0.254). The ORR was 25% for Child–Pugh B patients, which was numerically lower than the result of 29.4% for Child–Pugh A patients (*p* = 0.710) (RECIST v1.1) (Supplementary Table 1). Among all 71 patients, the median TTP was 11.2 months (95% CI, 5.1–17.3) according to RECIST v1.1 and 10.9 months (95% CI, 8.4–13.5) according to mRECIST. The TTP was 15.3 months (95% CI, 3.6–27.0) for patients in the first-line setting and 9.4 months (95% CI, 2.8–16.1) for patients with prior systemic therapy (RECIST v1.1).

### PFS and OS

**Fig. 1:**
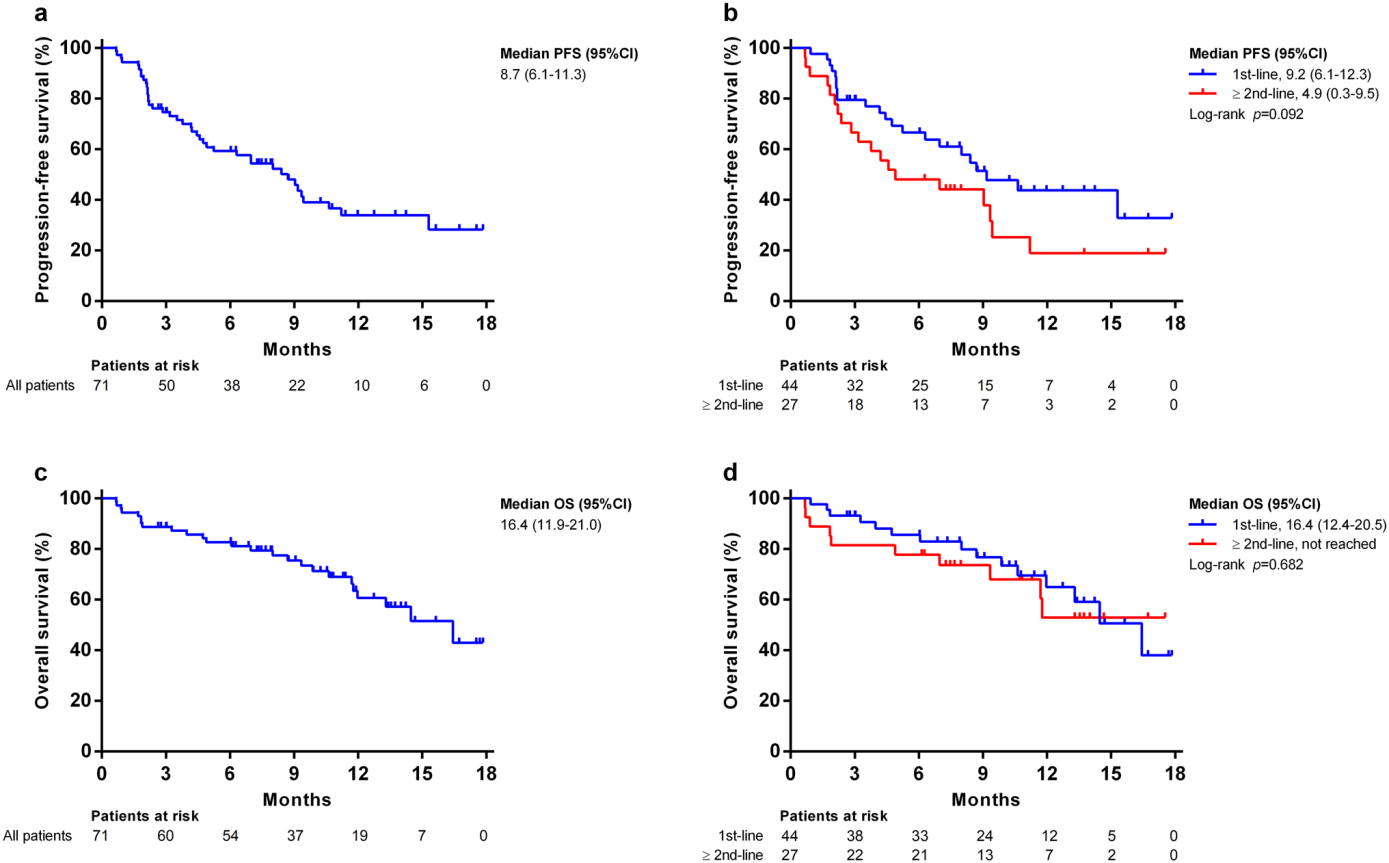
Progression-free survival (PFS) and overall survival (OS) of entire cohort of HCC patients. Kaplan–Meier estimates of PFS according to RECIST v1.1 **a** among all 71 patients **b** stratified by systemic therapy-naïve and -experienced patients; OS **c** among entire 71 patients and **d** stratified by systemic therapy-naïve and -experienced patients

The median PFS was 8.7 months (95% CI, 6.1–11.3 months) according to RECIST1.1 (Fig. 1a), 9.2 months (95% CI, 6.1–12.3 months) for patients in the first-line setting, and 4.9 months (95% CI, 0.3–9.5 months) for cases that experienced prior systemic therapy (RECIST v1.1, *p* = 0.092, Fig. 1b). The median OS was 16.4 months overall (95%CI, 11.9–21.0 months, Fig. 1c), 16.4 months (95% CI, 12.4–20.5) for patients in the first-line setting, and not estimable for patients with prior systemic therapy (RECIST v1.1, *p* = 0.682, Fig. 1d).

**Fig. 2:**
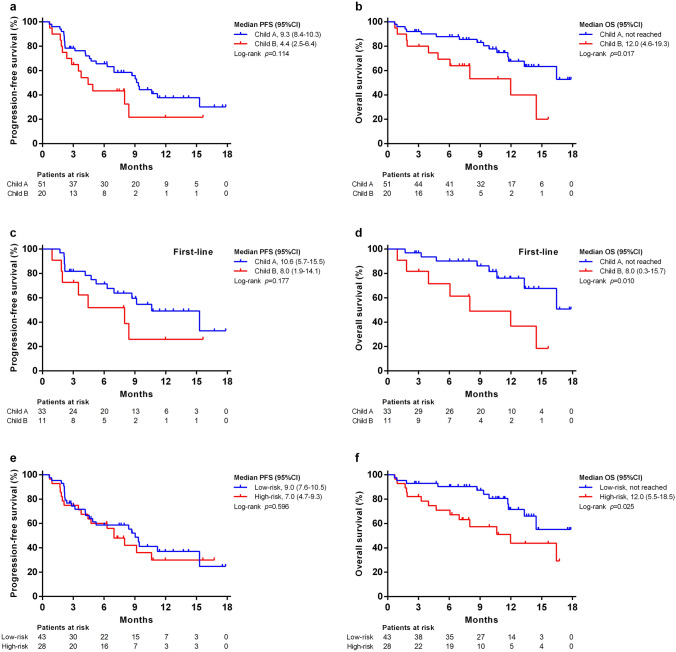
Progression-free survival (PFS) and overall survival (OS) based on Child–Pugh class and tumor risk. Kaplan–Meier estimates of **a** PFS according to RECIST v1.1 among all 71 patients; **b** OS among entire cohort; **c** PFS according to RECIST v1.1 in the first-line-setting patients; **d** OS in the first-line-setting patients; **e** PFS according to RECIST v1.1; **f** OS stratified by tumor risk

Compared to patients with Child–Pugh A, poorer median PFS was noted for Child–Pugh B patients (9.3 months vs. 4.4 months), but the difference was not statistically significant (Fig. 2a). Child–Pugh class also affected OS: patients with Child–Pugh A had significantly better median OS than those with Child–Pugh B (not reached vs. 12 months, *p* = 0.017) (Fig. 2b). Among the 44 patients in the first-line setting, the median PFS was 10.6 months (95% CI, 5.7–15.5) for patients with Child–Pugh A and 8.0 months (95% CI, 1.9–14.1) for patients with Child–Pugh B (RECIST v1.1, *p* = 0.177, Fig. 2c). The median OS was not estimable for patients with Child–Pugh A and 8.0 months (95% CI, 0.3–15.7) for patients with Child–Pugh B (RECIST v1.1, *p* = 0.010, Fig. 2d).

**Fig. 3:**
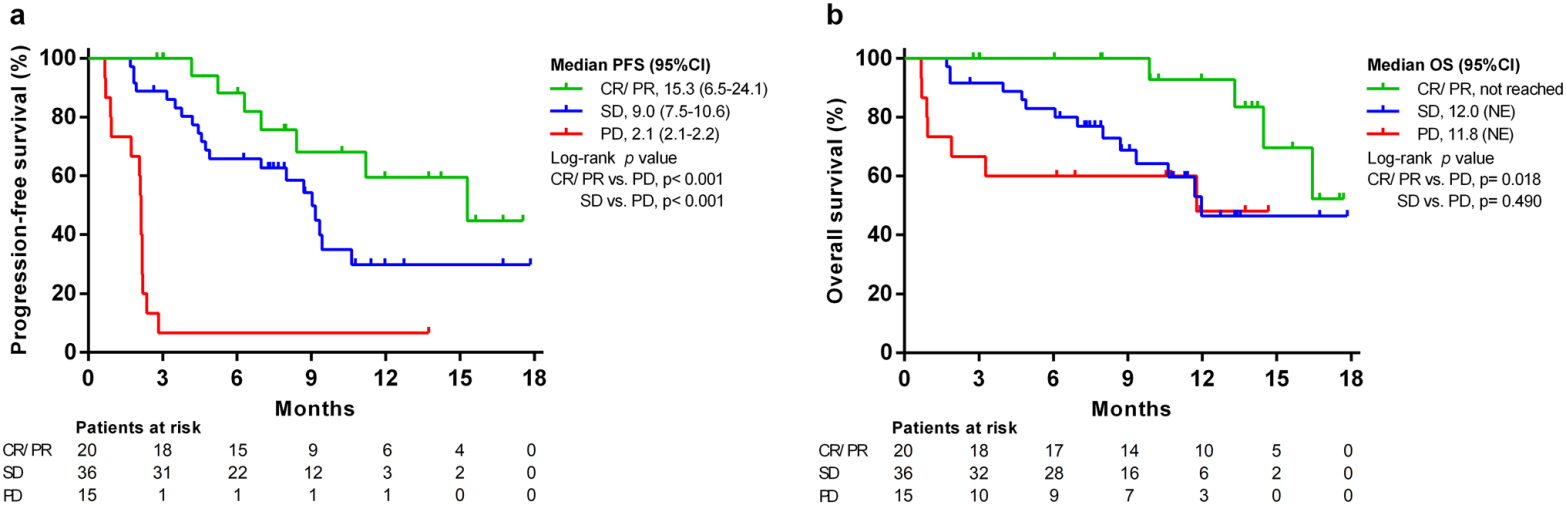
Progression-free survival (PFS) and overall survival (OS) according to the treatment response to lenvatinib plus pembrolizumab. Kaplan–Meier curves of **a** PFS according to RECIST v1.1; **b** OS stratified by treatment response. CR, complete response; PD, progressive disease; PR, partial response; SD, stable disease

Although the PFS (7.0 vs. 9.0 months, *p* = 0.596) was not significantly different between patients with high-risk and low-risk tumors (Fig. 2e), poorer OS was observed among patients with high-risk tumors (12 months vs. not reached, *p* = 0.025) in the Kaplan–Meier survival analysis (Fig. 2f). The PFS and OS of patients based on treatment responses are shown in Fig. 3a, b. Patients who achieved an objective response had significantly better PFS (15.3 vs. 2.1 months, *p* < 0.001) and OS (Not reached vs. 11.8 months, *p* = 0.018) than PD patients.

### Early AFP response

For the 53 patients with baseline AFP ≥ 10 ng/ml, 42 (79.2%) had AFP reduction within 4 weeks with a median AFP change to − 43.6%. Interestingly, both patients with and without objective response according to RECIST v1.1 had significant AFP reduction (median: − 48.6% vs. − 36.3%, *p* = 0.456). There was no significant difference in ORR (33.3% vs. 28.6%, *p* = 1.000) and DCR (87.2% vs. 71.4%, *p* = 0.222) between patients with and without early AFP reduction (> 10%) [25].

### ALBI score changes and safety profiles

**Fig. 4:**
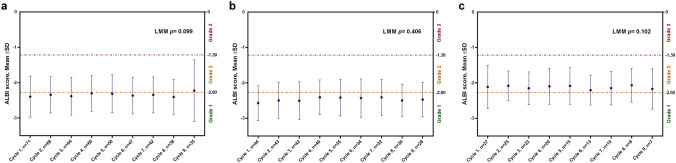
ALBI score changes during the treatment course. Change in mean ALBI score among **a** entire cohort patients (*n* = 71); **b** systemic therapy-naïve subgroup (*n* = 44); and **c** systemic therapy-experienced subgroup (*n* = 27). ALBI, albumin–bilirubin; SD, standard deviation

The median number of cycles of pembrolizumab was 7 cycles (range, 1–26 cycles), and the median dose of lenvatinib was 84.7% of the standard dose (interquartile range, 68.7% to 100%). Figure 4a shows the changes in ALBI score among patients who received lenvatinib plus pembrolizumab according to a linear mixed model. The mean ALBI score remained stable during the treatment course (*p* = 0.099). In addition, the ALBI score in the systemic therapy-naïve subgroup (*n* = 44) and systemic therapy-experienced subgroup (*n* = 27) both showed no significant change with respect to the treatment cycles (*p* = 0.406, Fig. 4b; *p* = 0.102 Fig. 4c).

TRAEs occurred in 97.2% (69/71) of patients (Supplementary Table 2), including 27 (96.4%) of the systemic therapy-naïve patients and 42 (97.7%) of the systemic therapy-experienced cases. The most common any-grade TRAE was fatigue (56.3%), followed by hypertension (49.3%), palmer-plantar syndrome (46.5%), decreased appetite (40.8%), and hypothyroidism (38%). The most common AEs with grade ≧ 3 were hepatitis (7%), hypertension (4.2%), decreased appetite (4.2%), and skin psoriasis flare (4.2%). Variceal bleeding was noted in 4 patients. Dose reduction of lenvatinib occurred in 20 (28.2%) cases.

AEs led to interruption of pembrolizumab treatment in 9 (12.7%) cases, and 29 (40.8%) cases used steroids due to an impression of immune-related adverse events. Of the 29 cases that used steroid treatment, 10 cases received IV methylprednisolone (0.5–1 mg/kg/day) for grade 3/4 immune-related adverse events and grade 2 pneumonitis. The other 19 patients received a low-dose (< 10 mg/day) oral form of prednisolone for skin rash, pruritus, and grade 1/2 hepatitis. Discontinuation of the combination treatment because of TRAEs was noted in 3 (4.2%) patients (1 case of colitis and 2 cases of pneumonitis). In general, patients with prior systemic therapy did not have significantly higher risk of any grade of TRAE than patients in the first-line setting except for a higher chance of pruritus (44.4% vs. 15.9%) in patients with prior systemic therapy. The occurrence rate of grade 3/4 TRAEs was 35.7% in patients with high-risk tumors (18.6% in patients with low-risk tumors, *p* = 0.105) and 35% in patients with Child–Pugh B (21.6% in patients with Child–Pugh A, *p* = 0.242) (Supplementary Table 3).

### Factors associated with PFS and OS

**Fig. 5:**
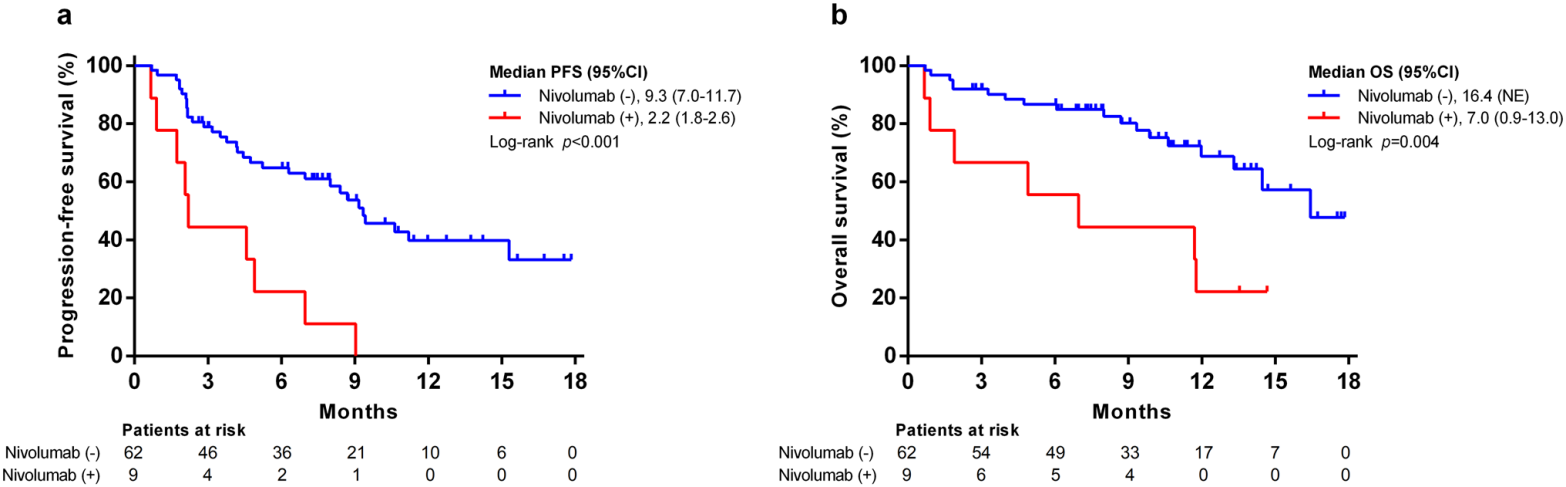
Prior nivolumab treatment associated with worse progression-free survival (PFS) and overall survival (OS). Kaplan–Meier curves of **a** PFS according to RECIST v1.1; **b** OS stratified by nivolumab experience

In the univariate analysis, ECOG status ≥ 1, INR > 1.2, and prior nivolumab experience were factors associated with PFS according to RECIST v1.1. In the multivariate analysis, previous experience with nivolumab (HR = 3.253, 95% CI: 1.473 to 7.183, *p* = 0.004) was the only independent risk factor for PFS (Supplementary Table 4 and Fig. 5a). Factors associated with OS in the univariate analysis were ECOG status ≧ 1, bile duct involvement, Child–Pugh class B, and prior systemic therapy using nivolumab. In the multivariate analysis, Child–Pugh class B (HR = 2.646, 95% CI: 1.053 to 6.651) and previous nivolumab experience (HR = 3.340, 95% CI:1.277 to 8.734) were significant prognostic factors for poorer OS (Table 3 and Fig. 5b). Among patients naïve to nivolumab, the PFS and OS of those with MKI experience were 9.4 months (vs. 9.2 months, *p* = 0.772) and not reached (vs. 16.4 months, *p* = 0.261), respectively, in comparison to those in the first-line setting (Supplementary Fig. 1).

**Table 3:** Univariate and multivariate analyses for factors associated with overall survival

Characteristics		Univariate analysis			Multivariate analysis		
		HR	95% CI	*p* Value	HR	95% CI	*p* Value
Age (yrs)	> 60 versus ≤ 60	1.797	0.773–4.175	0.173			
Gender	Male versus female	1.091	0.326–3.650	0.887			
ECOG ≥ 1	Yes versus no	2.78	1.226–6.302	0.014	1.889	0.774–4.608	0.162
Etiology (viral hepatitis)	Yes versus no	0.6	0.258–1.400	0.237			
Tumor number	Multiple versus single	0.782	0.336–1.818	0.568			
Tumor ≥ 50% liver volume	Yes versus no	1.955	0.833–4.586	0.123			
Main portal vein invasion	Yes versus no	1.049	0.393–2.798	0.923			
Bile duct involvement	Yes versus no	6.002	1.338–26.934	0.019	2.525	0.524–12.173	0.249
Extrahepatic metastasis	Yes versus no	0.621	0.274–1.408	0.254			
BCLC stage	Stage C versus B	1.089	0.408–2.906	0.865			
AFP ng/mL	> 400 versus ≤ 400	2.16	0.952–4.902	0.065	2.219	0.950–5.187	0.066
NLR	> 2.5 versus ≤ 2.5	1.179	0.468–2.969	0.727			
	> 5 versus ≤ 5	1.832	0.735–4.566	0.194			
INR	> 1.2 versus ≤ 1.2	0.575	0.240–1.382	0.216			
Platelet count	> 100 versus ≤ 100	1.308	0.490–3.492	0.592			
ALT, U/L	> 40 versus ≤ 40	0.816	0.370–1.798	0.614			
AST, U/L	> 40 versus ≤ 40	1.222	0.523–2.853	0.643			
Child–Pugh class	Class B versus A	2.629	1.153–5.994	0.021	2.646	1.053–6.651	0.039
ALBI grade	m2b/ 3 versus 1/m2a	1.837	0.836–4.037	0.13			
Fib-4 score	> 6.5 versus ≦ 6.5	1.338	0.558–3.207	0.514			
Systemic treatment	≧ 2nd line versus 1st line	1.182	0.531–2.635	0.682			
MKI experience	Yes versus No	1.205	0.531–2.735	0.655			
Nivolumab experience	Yes versus no	3.335	1.381–8.055	0.007	3.34	1.277–8.734	0.014
Early AFP response (≥ 10%)^a^	Yes versus no	1.633	0.472–5.650	0.438			
Early AFP response (any)^b^	Yes versus no	1.164	0.337–4.023	0.81			

53 patients with baseline AFP ≥ 10 ng/ml were analyzed

*AFP* alpha-fetoprotein, *ALT* alanine aminotransferase, *AST* aspartate aminotransferase, *BCLC* barcelona clinic liver cancer, *CI* confidence interval, *ECOG* eastern cooperative oncology group, *INR* international normalized ratio, *MKI* multikinase inhibitor, *NLR* neutrophil-to-lymphocyte ratio

^a^AFP reduction ≥ 10% within 4 weeks according to 10–10 rule

^b^AFP reduction in any degree within 4 weeks

### Role of PD-L1 expression in tumor response and survival

**Fig. 6:**
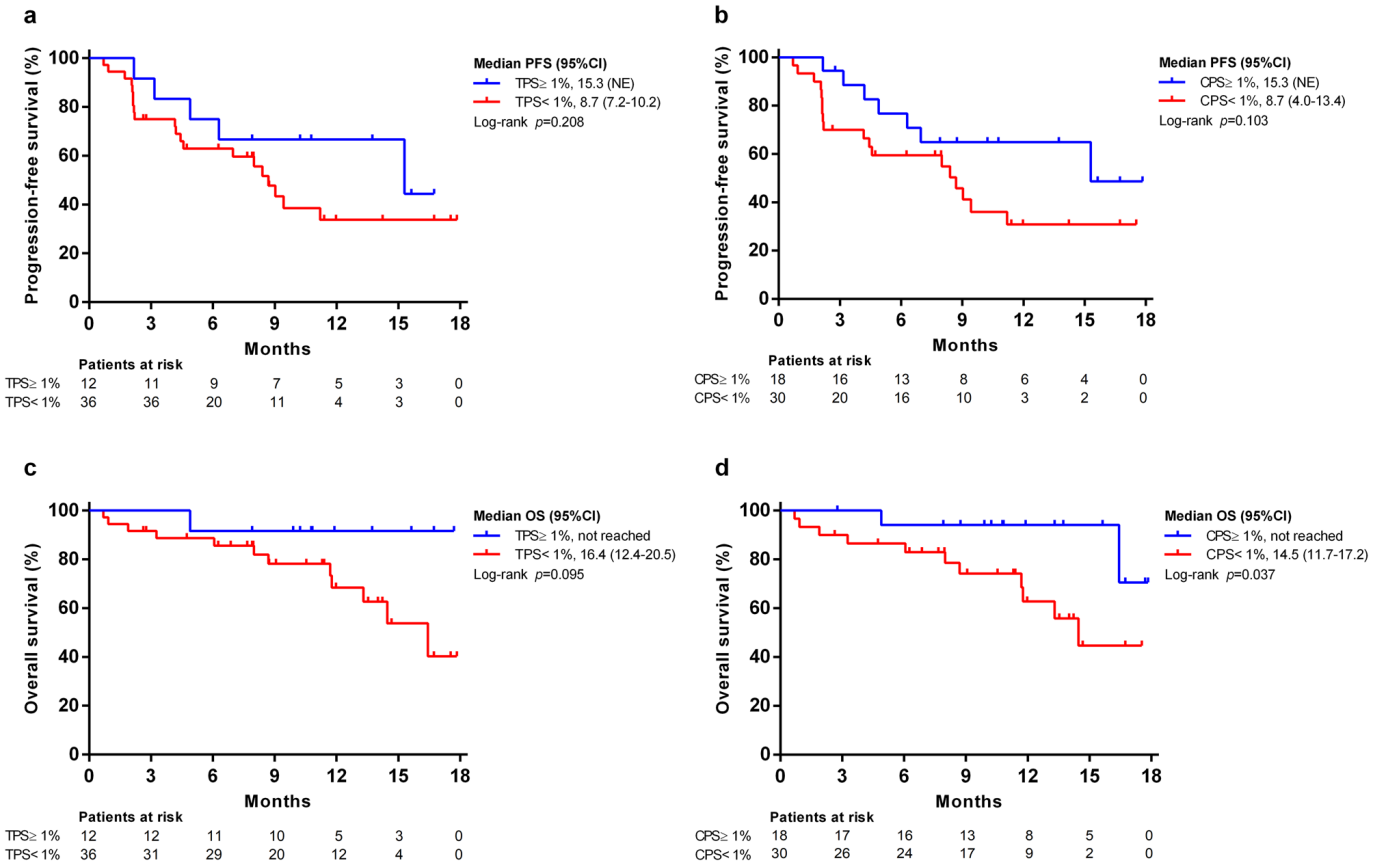
Association of PD-L1 with progression-free survival (PFS) and overall survival (OS). Kaplan–Meier curves of PFS according to RECIST v1.1 stratified by **a** TPS ≥ 1% or < 1%; **b** CPS ≥ 1% or < 1%; OS stratified by **c** TPS ≥ 1% or < 1%; **d** CPS ≥ 1% or < 1%. CPS, combined positive score; TPS, tumor proportion score

There were 48 patients with available liver-tumor tissues for the determination of PD-L1 expression (25 archival specimens and 23 freshly derived specimens). In general, the ORR was higher in TPS-positive cases (58.3% vs. 25%, *p* = 0.073) and CPS-positive cases (50% vs. 23.3%, *p* = 0.058) based on RECIST v1.1 (Supplementary Table 5). The median PFS for TPS-positive and negative patients were 15.3 months and 8.7 months (*p* = 0.208, Fig. 6a), while those for CPS-positive and negative patients were 15.3 months and 8.7 months (*p* = 0.103, Fig. 6b), respectively. The median OS for TPS-positive and negative patients were not reached and 16.4 months (TPS, *p* = 0.095, Fig. 6c), while those for CPS-positive and negative patients were not reached and 14.5 months (CPS, *p* = 0.037, Fig. 6d), respectively.

## Discussion

To the best of our knowledge, there has been no study comparing lenvatinib plus pembrolizumab in the first-line setting and systemic-experienced uHCC and in Child–Pugh B patients. In our study, we found that the treatment-related adverse events from lenvatinib plus pembrolizumab were similar between systemic therapy-naïve and -experienced uHCC patients, but the PFS and OS are compromised in patients with prior systemic therapy, particularly in patients with prior nivolumab treatment failure. Our findings provide important information on lenvatinib plus pembrolizumab for uHCC patients with high-risk tumors, Child–Pugh class B, and prior systemic treatment exposure. For the responders, the response was also enduring.

The ORR and DCR of lenvatinib plus pembrolizumab were generally better than those of MKI or ICI monotherapy [8, 13, 14]. But for patients with prior systemic therapy, the ORR of lenvatinib plus pembrolizumab (18.5% according to RECIST v1.1) was similar to that of lenvatinib (18.8% in REFLECT study) or pembrolizumab monotherapy (18.3% in KEYNOTE-240 study) [8, 14]. This finding indicated that a certain degree of MKI and ICI resistance might exist after prior systemic therapy. Nevertheless, the DCR of lenvatinib plus pembrolizumab was still high across systemic-naïve and -experienced cases.

There has been no applicable baseline factor to predict the response to ICI immunotherapy for HCC in previous studies [25]. In this study, prior nivolumab failure was the only factor significantly associated with PFS. This finding implies that prior anti-PD-1 treatment may have a detrimental effect on repeated anti-PD-1 immunotherapy by blocking the same immune checkpoint.

Our previous real-world cohort study has demonstrated that liver reserve (Child–Pugh class A) determined overall survival on ICI therapy [25]. In this study, Child–Pugh class B and previous nivolumab experience were risk factors for OS. Previous studies suggest that MKI could enhance the response to ICI immunotherapy [29], but the current study showed that prior MKI treatment did not have a positive effect on the response to lenvatinib plus pembrolizumab. Atezolizumab plus bevacizumab is still being developed as a first-line treatment for advanced HCC, so the outcomes of this treatment followed by lenvatinib/pembrolizumab still require further exploration.

Most clinical trials excluded patients with high-risk tumors [8, 13, 14, 18]. Tumor burden ≧ 50% liver volume, main portal vein invasion, and bile duct involvement were not factors associated with PFS and OS in this study. This indicates that patients with high-risk tumors are still potential candidates for lenvatinib plus pembrolizumab treatment, but only if their liver function is well preserved. In addition, our study confirmed that there was no negative effect on ALBI score during lenvatinib plus pembrolizumab treatment.

Although nearly all patients experienced adverse events, only 3 patients discontinued the treatment due to such events. No unexpected adverse effects were discovered, and the incidence rates of TRAEs were similar in the therapy-naïve and -experienced patients. Only a higher frequency of pruritus was observed in patients with prior systemic therapy. AEs with grade ≧ 3 were rare, including pneumonitis, psoriasis, hypertension, diarrhea, and hepatitis.

We previously proposed a “10–10 rule” based on early AFP response to predict ORR and survival of ICI monotherapy in advanced HCC [25]. But AFP reduction is common when using lenvatinib/pembrolizumab combinations, AFP reduction could happen in patients without objective response, and early AFP reduction did not have a predictive role for lenvatinib/pembrolizumab combinations. The predictive value of PD-L1 expression in response to ICI for HCC has not yet been confirmed [12, 13, 30]. A positive result for PD-L1 expression has been associated with significantly better OS and PFS [31, 32]. In the present study, CPS-positive cases had a trend of higher ORR and better median OS than CPS-negative patients. These findings suggest that PD-L1 expression has a potential role in selecting candidates for lenvatinib plus pembrolizumab treatment for uHCC.

There are several limitations to this study. First, there was no control arm for comparison. However, the ORR and DCR of lenvatinib plus pembrolizumab were unequivocally higher than in previous studies on ICI and MKI monotherapy. Second, the case number was not large, although this has been the largest prospective real-world study on lenvatinib plus pembrolizumab for unresectable HCC so far. Nevertheless, our findings highlight the potential for future expansion of indications for lenvatinib plus pembrolizumab for uHCC patients with high-risk tumors, Child–Pugh B, or previous MKI experience.

In conclusion, the real-world data on lenvatinib plus pembrolizumab showed comparable responses and survival to an early phase clinical trial without unexpected adverse effects, even in patients with high-risk tumors and Child–Pugh B status.

### Supplementary Information

Below is the link to the electronic supplementary material.

Supplementary file1 (PDF 392 KB)

## Data Availability

The dataset used for this study is available from the corresponding author upon reasonable request.

## Abbreviations

AASLD: American association for the study of liver disease
AFP: Alpha-fetoprotein
ALT: Alanine aminotransferase
AST: Aspartate aminotransferase
BCLC: Barcelona-clinic-liver-cancer
CI: Confidence interval
CR: Complete response
CPS: Combined positive score
CT: Computed tomography
DCR: Disease control rate
DOR: Duration of response
ECOG: Eastern cooperative oncology group
HBsAg: Hepatitis B surface antigen
HBV: Hepatitis B virus
HCC: Hepatocellular carcinoma
HCV: Hepatitis C virus
ICI: Immune check point inhibitor
INR: International normalized ratio
MKI: Multikinase inhibitor
MRI: Magnetic resonance image
NLR: Neutrophil-to-lymphocyte ratio
NUCs: Nucleos/tide analogues
OR: Odds ratio
ORR: Objective response rate
OS: Overall survival
PD: Progressive disease
PD-(L)1: Programmed death (ligand) 1
PFS: Progression-free survival
PR: Partial response
(m)RECIST: Modified response evaluation criteria in solid tumors
TACE: Transarterial chemoembolization
TFDA: Taiwan food and drug administration
TPS: Tumor proportion score
TTP: Time to progression
TTR: Time to response
TRAE: Treatment-related adverse effects
v1.1: Version 1.1
VEGF(R): Vascular endothelial growth factor (receptor)
