# Supplementary material for: Lenvatinib plus pembrolizumab for systemic therapy-naïve and -experienced unresectable hepatocellular carcinoma
Source: Cancer Immunol Immunother. 2022 Mar 28;71(11):2631–43. doi: 10.1007/s00262-022-03185-6 (PMC9519717; doi:10.1007/s00262-022-03185-6)

Supplementary Table 1. Best objective response evaluated by RECIST1.1 in different Child-Pugh class and tumor risk subgroups

	Child-Pugh class			Tumor risk		
Parameter, n(%)	Child A (n=51)	Child B (n=20)	*p* Value	Low-risk tumors (n=43)	High-risk tumors (n=28)	*p* Value
CR	1(2)	0(0)	1.000	1(2.3)	0(0)	1.000
PR	14(27.5)	5(25)	0.834	12(27.9)	7(25)	0.787
SD	26(51)	10(50)	0.941	19(44.2)	17(60.7)	0.173
PD	10(19.6)	5(25)	1.000	11(25.6)	4(14.3)	0.181
ORR	15(29.4)	5(25)	0.710	13(30.2)	7(25)	0.632
DCR	41(80.4)	15(75)	0.748	32(74.4)	24(85.7)	0.254

CR, complete response; DCR, disease control rate; ORR, objective response rate; PD, progressive disease; PR, partial response; SD, stable disease.

Supplementary Table 2. Treatment related adverse events

Symptom, n(%)	Any grade				Grade 3 or 4		
	All patients (n=71)	First-line setting (n=44)	Systemic therapy-experienced (n=27)	*p* Value	All patients (n=71)	First-line setting (n=44)	Systemic therapy-experienced (n=27)
Fatigue	40(56.3)	25(56.8)	15(55.6)	0.917	1(1.4)	1(2.3)	0(0)
Hypertension	35(49.3)	25(56.8)	10(37)	0.106	3(4.2)	1(2.3)	1(3.7)
Palmar-plantar syndrome	33(46.5)	24(54.5)	9(33.3)	0.082	1(1.4)	1(2.3)	0(0)
Decreased appetite	29(40.8)	19(43.2)	10(37)	0.609	3(4.2)	2(4.5)	1(3.7)
Hypothyrodism	27(38)	20(45.5)	7(25.9)	0.100	0(0)	0(0)	0(0)
Diarrhea	20(28.2)	11(25)	9(33.3)	0.449	2(2.8)	1(2.3)	1(3.7)
Pruritis	19(26.8)	7(15.9)	12(44.4)	0.008	0(0)	0(0)	0(0)
Laryngeal inflammation	18(25.4)	13(29.5)	5(18.5)	0.300	0(0)	0(0)	0(0)
Proteinuria*	17(25.8)	12(30)	5(19.2)	0.328	0(0)	0(0)	0(0)
Hepatitis	8(11.3)	5(11.4)	3(11.1)	0.974	5(7)	3(6.8)	2(7.4)
Poriasis	3(4.2)	3(6.8)	0(0)	0.283	3(4.2)	3(6.8)	0(0)
Pneumonitis	3(4.2)	3(6.8)	0(0)	0.283	2(2.8)	2(4.5)	0(0)

*Available data for 66 patients, including 40 patients in first-line setting.

Supplementary Table 3. Treatment related adverse events in different Child-Pugh class and tumor risk subgroups

Symptom, n(%)	Child-Pugh class				Tumor risk			
	Any grade		Grade 3 or 4		Any grade		Grade 3 or 4	
	Child A (n=51)	Child B (n=20)	Child A (n=51)	Child B (n=20)	Low-risk tumor (n=43)	High-risk tumor (n=28)	Low-risk tumor (n=43)	High-risk tumor (n=28)
Fatigue	30(58.8)	10(50)	0(0)	1(5)	23(53.5)	17(60.7)	0(0)	1(3.6)
Hypertension	26(51)	9(45)	2(3.9)	1(5)	20(46.5)	15(53.6)	3(7)	0(0)
Palmar-plantar syndrome	26(51)	7(35)	1(2)	0(0)	24(55.8)	9(32.1)	0(0)	1(3.6)
Decreased appetite	20(39.2)	9(45)	1(2)	2(10)	15(34.9)	14(50.0)	0(0)	3(10.7)
Hypothyrodism	19(37.3)	8(40)	0(0)	0(0)	15(34.9)	12(42.9)	0(0)	0(0)
Diarrhea	16(31.4)	4(20)	1(2)	1(5)	13(30.2)	7(25)	1(2.3)	1(3.6)
Pruritis	16(31.4)	3(15)	0(0)	0(0)	13(30.2)	6(21.4)	0(0)	0(0)
Laryngeal inflammation	13(15.5)	5(25)	0(0)	0(0)	8(18.6)	10(35.7)	0(0)	0(0)
Proteinuria*	12(26.1)	5(25)	0(0)	0(0)	10(25.6)	7(25.9)	0(0)	0(0)
Hepatitis	6(11.8)	2(10)	3(5.9)	2(10)	5(11.6)	3(10.7)	2(4.7)	3(10.7)
Poriasis	2(3.9)	1(5)	2(3.9)	1(5)	2(4.7)	1(3.6)	2(4.7)	1(3.6)
Pneumonitis	2(3.9)	1(5)	1(2)	1(5)	2(4.7)	1(3.6)	1(2.3)	1(3.6)

Supplementary Table 4. Univariate and multivariate analyses for factors associated with progression free survival based on RECIST1.1

Characteristics		Univariate Analysis			Multivariate Analysis Model 1			Multivariate Analysis Model 2		
		HR	95% CI	*p* Value	HR	95% CI	*p* Value	HR	95% CI	*p* Value
Age(yr)	>60 vs. ≤60	1.544	0.816-2.923	0.182						
Gender	Male vs. Female	1.282	0.503-3.272	0.603						
ECOG≥1	Yes vs. No	1.916	1.022-3.591	0.043	1.628	0.848-3.124	0.143	1.542	0.803-2.962	0.193
Etiology (Viral hepatitis)	Yes vs. No	0.625	0.317-1.234	0.176						
Tumor number	Multiple vs. single	0.934	0.476-1.833	0.843						
Tumor ≥50% liver volume	Yes vs. No	0.941	0.447-1.977	0.872						
Main portal vein invasion	Yes vs. No	0.881	0.406-1.909	0.881						
Bile duct involvement	Yes vs. No	2.225	0.530-9.340	0.274						
Extrahepatic metastasis	Yes vs. No	0.976	0.525-1.815	0.939						
BCLC stage	Stage C vs. B	0.990	0.457-2.145	0.980						
AFP, ng/mL	>400 vs. ≤400	1.321	0.714-2.445	0.375						
NLR	>2.5 vs. ≤2.5	0.965	0.470-1.979	0.922						
INR	>1.2 vs. ≤1.2	0.546	0.282-1.057	0.073	0.537	0.275-1.050	0.069	0.617	0.315-1.206	0.158
Platelet count	>100 vs. ≤100	0.581	0.295-1.143	0.116						
ALT, U/L	>40 vs. ≤40	0.622	0.334-1.158	0.134						
AST, U/L	>40 vs. ≤40	0.662	0.353-1.244	0.200						
Child–Pugh class	Class B vs. A	1.701	0.873-3.317	0.119						
ALBI grade	m2b/ 3 vs. 1/ m2a	1.111	0.593-2.083	0.742						
Fib-4 score	>6.5 vs. ≤6.5	1.514	0.771-2.973	0.228						
Systemic treatment	≥2nd line vs.1st line	1.687	0.912-3.121	0.096	1.651	0.872-3.127	0.124			
MKI experience	Yes vs. No	1.475	0.791-2.747	0.221						
Nivolumab experience	Yes vs. No	3.966	1.846-8.518	<0.001				3.253	1.473-7.183	0.004
Early AFP response (≥10%)[a]	Yes vs. No	1.099	0.469-2.577	0.828						
Early AFP response (any)[b]	Yes vs. No	0.681	0.290-1.602	0.379						

[a]53 patients with baseline AFP≥10 ng/ml were analyzed; [a] AFP reduction ≥10% within 4 weeks according to 10-10 rule. [b] AFP reduction in any degree within 4 weeks.

AFP, alpha-fetoprotein; ALT, alanine aminotransferase; AST, aspartate aminotransferase; BCLC, Barcelona-Clinic-Liver-Cancer; CI, confidence interval; ECOG, Eastern Cooperative Oncology Group; INR, international normalized ratio; MKI, multikinase inhibitor; NLR, neutrophil-to-lymphocyte ratio.

Supplementary Table 5. Association of the expression level of Programmed death-ligand 1(PD-L1) with clinical outcomes[a]

	Best objective response[b]			Progression free survival[b] (≥1% vs. <1%)			Overall survival (≥1% vs. <1%)		
	<1%	≥1%	*p* value	HR	95% CI	*p* Value[†]	HR	95% CI	*p* Value[c]
Combined positive score	7(23.3%)	9(50%)	0.058	0.489	0.203-1.177	0.110	0.226	0.049-1.032	0.055
Tumor proportion score	9(25%)	7(58.3%)	0.073	0.537	0.200-1.439	0.216	0.208	0.027-1.599	0.131

[a]Of 48 patients with available PD-L1 expression data. [b]Best objective response and progression-free survival are reviewed by RECIST v1.1. [c]p values are for the association of PD-L1 expression with overall survival and progression-free survival by use of Cox regression.

Supplementary Figure 1. Kaplan-Meier curves of patients with nivolumab +/- MKI or MKI alone treatment experienced compared with patients received lenvatinib plus pembrolizumab as first-line treatment. (a) progression-free survival [PFS] by RECIST v1.1 (b) Overall survival [OS]

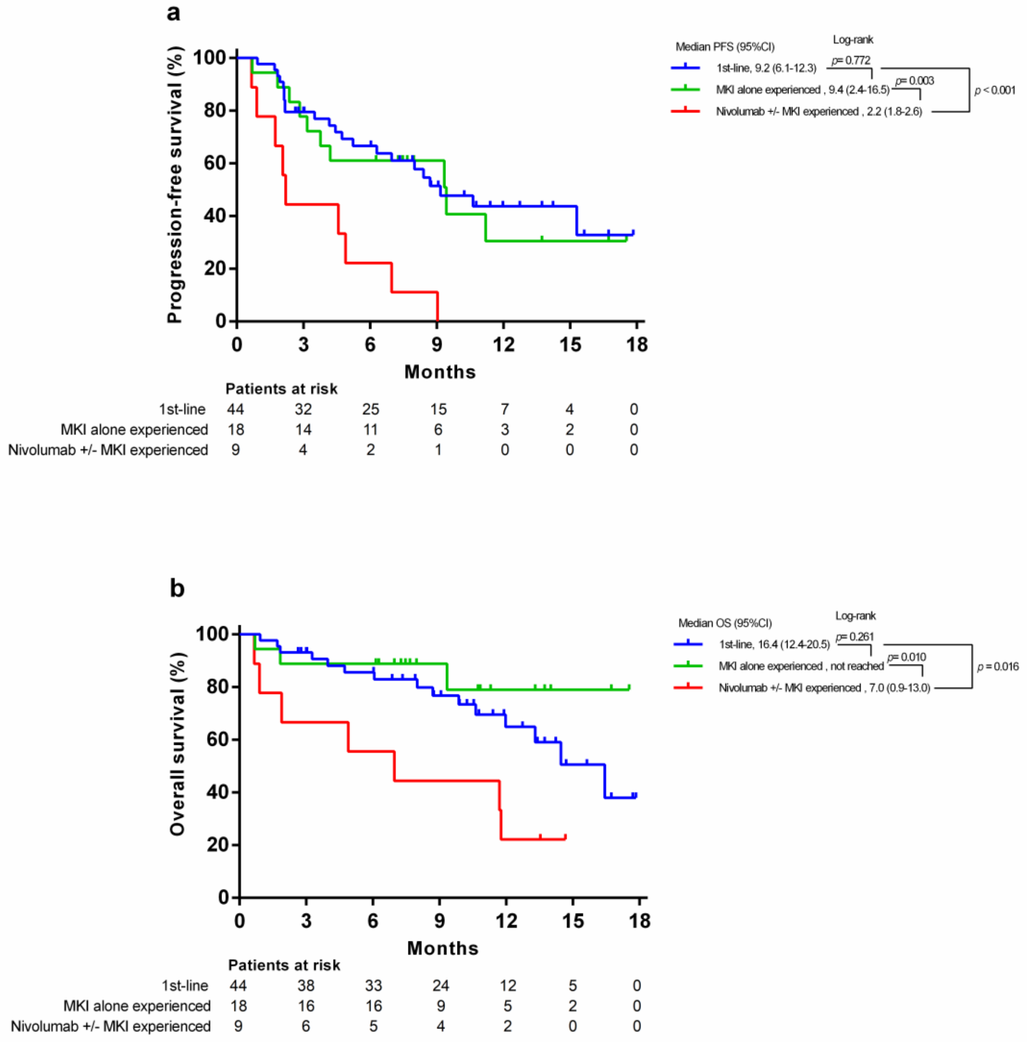

Supplement: Supplementary file 1 — Supplementary file1 (PDF 392 KB) [file 262_2022_3185_MOESM1_ESM.pdf]
